# Special Convergence of the Eyeballs in Severe Cases of Shock

**Published:** 1919-01

**Authors:** 


***Special Convergence of the Eyeballs in Severe Cases of Shock.*** By P. DESCOMPS, J. EUZIÈRE and P. MERLE. *La Presse Médicale*, October 3, 1918.

This special convergence has been detected by the authors in the course of the systematic equilibration trials practised in all the cases of shock with which they have been dealing. This convergence appears most distinctly during the rotatory tests; but it may also be detected after some other exciting cause, electric tests for instance.

After ten turns of the platform, it is stopped abruptly and the patient opens his eyes. In typical cases, the eyeballs converge most markedly, as in cases of strong inward squinting. This abnormal feature may last as long as one minute and a half, then gradually fades away, and both axes regain their normal direction. The phenomenon is not necessarily symmetrical; one of the eyeballs may be squinting inward more than the other, or may regain its normal position before the other.

Sometimes this convergence can only be detected by testing for associated lateral movements after preliminary rotatory tests. Both axes of the eyes do not remain strictly parallel. One of the eyes lags. This symptom seems to be connected with some disturbance of the extrinsic motricity: paresis of one or several of the muscles.

Finally, a true paralysis of the 6th pair of nerves may be observed in more severe cases. It is marked by a convergence of the eyeballs, following the lateral movements, without the necessity for the preliminary rotatory tests.

That convergence, whether bilateral and symmetrical, or unilateral, always proves, on the whole, some disequilibrium in the adductor muscles, exaggerated by the excitation due to the rotation of the platform.

The symptom seems to be due to slight lesions injuring either the motor centers in the brain, the centers of association or the connecting nerves.

The symptom of convergence does not seem to be directly connected with nystagmus; however, both phenomena may exist concomitantly.

